# Multilayer Polymer Photonic Aegises Against Near-Infrared Solar Irradiation Heating

**DOI:** 10.1021/acsami.1c25037

**Published:** 2022-03-19

**Authors:** Andrea Lanfranchi, Heba Megahd, Paola Lova, Davide Comoretto

**Affiliations:** Dipartimento di Chimica e Chimica Industriale, Università degli Studi di Genova, Via Dodecaneso 31, 16146, Genoa, Italy

**Keywords:** polymer photonic crystals, thermal shielding, solar irradiation, heating, near-infrared, dielectric mirror, sustainability

## Abstract

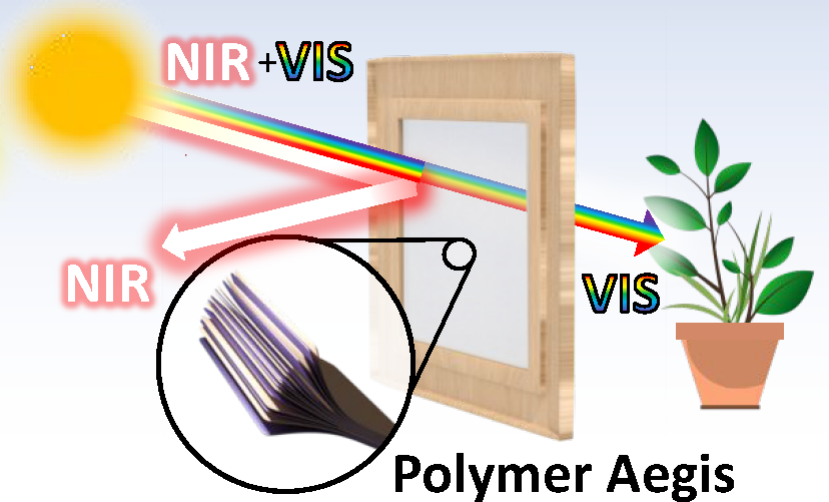

Preventing solar heating is nowadays of paramount interest in energy savings and health preservation. For instance, in building thermalization solar heating consumes an excess of energy leading to harmful CO_2_ emissions, while in food and beverage packaging it may lead to variation of organoleptic properties or even health issues. The phenomenon is attributed to the large presence of moieties with highly absorbing vibrational overtones and combination bands in the near-infrared spectral region that induces heating in water, moisture, and in polymers used in packaging. Thus, reducing and controlling the light absorbed by these materials with effective low-cost passive systems can play a major role in energy saving and health preservation. In this work, different polymer dielectric mirrors are reported, made of poly(*N*-vinylcarbazole) and either cellulose acetate or poly(acrylic acid), and able to selectively reflect near-infrared radiation while maintaining high transparency in the visible range. To this end, simple, tandem, and superperiodic mirrors are used to shield radiation impinging on samples of water and paraffin, demonstrating shielding efficiencies up to 52% with respect to unshielded references, promising a new paradigm to solve thermal management issues.

## 1 Introduction

Year by year, the looming ghost of climate change polarizes the international attention toward sustainability, energy efficiency, and savings, as also indicated by the 12 principles of sustainable chemistry,^1^ the United Nations 2030 agenda for sustainable development,^2^ and by the European Green Deal.^3^ A general problem in the field is related to undesirable and excessive radiative heating by sunlight, which especially affects hot and sunlit regions and causes high emissions by air conditioning systems. As an example, in the southeastern United States, air conditioning corresponds to 27% of the total energy expenditure of homes.^4^ In India, a 25% increase in total CO_2_ emissions can be expected by 2100 exclusively due to the installation of air conditioning in buildings.^5^ Similar trends are also expected for other developing countries such as Brazil.^6^ This is an issue in transportation as well, where air conditioning usage means consumption of fuel and subsequent carbon dioxide emissions.^7^ Excessive sunlight heating also affects agriculture, when overheating in greenhouses during the summer season reduces crop yields and increases water consumption.^8,9^ Even the food and drink industry suffers from this problem. For instance, when heated during transport or displaying, plastic containers release small amounts of substances within their contents, possibly altering their organoleptic properties in the long run^10^ and inducing uncontrolled microbial growth.^11,12^

Approaches to deal with this problem include, for instance, radiative cooling, which exploits materials whose thermal emission is concentrated in the atmospheric transparency window (8–13 μm). This way, the emitted blackbody radiation gets transmitted through the atmosphere and then to the cold emptiness of space, maximizing the heat loss.^13,14^ Radiative cooling, however, needs a strong emitter to be used in conjunction with an efficient mirror, to reflect all the radiation which would cause heating in the first place.^13,14^ Nonetheless, the possibility of light reflection to reduce heating is interesting on its own. Commercially employed approaches exploit the addition of highly reflecting and/or scattering particles to paints. For instance, it has been reported that adding TiO_2_ to a white painted surface leads to reflecting up to 85% of sunlight, and thus to excellent thermal shielding.^15^ However, this approach does not solve issues related to modern buildings and skyscrapers, which are entirely covered by windows that must remain transparent to the eye. Using thin inorganic films on windows could be a viable solution in that case, but poor wavelength tuning and the high costs of deposition on large surfaces are hindering their use.

**Figure 1:**
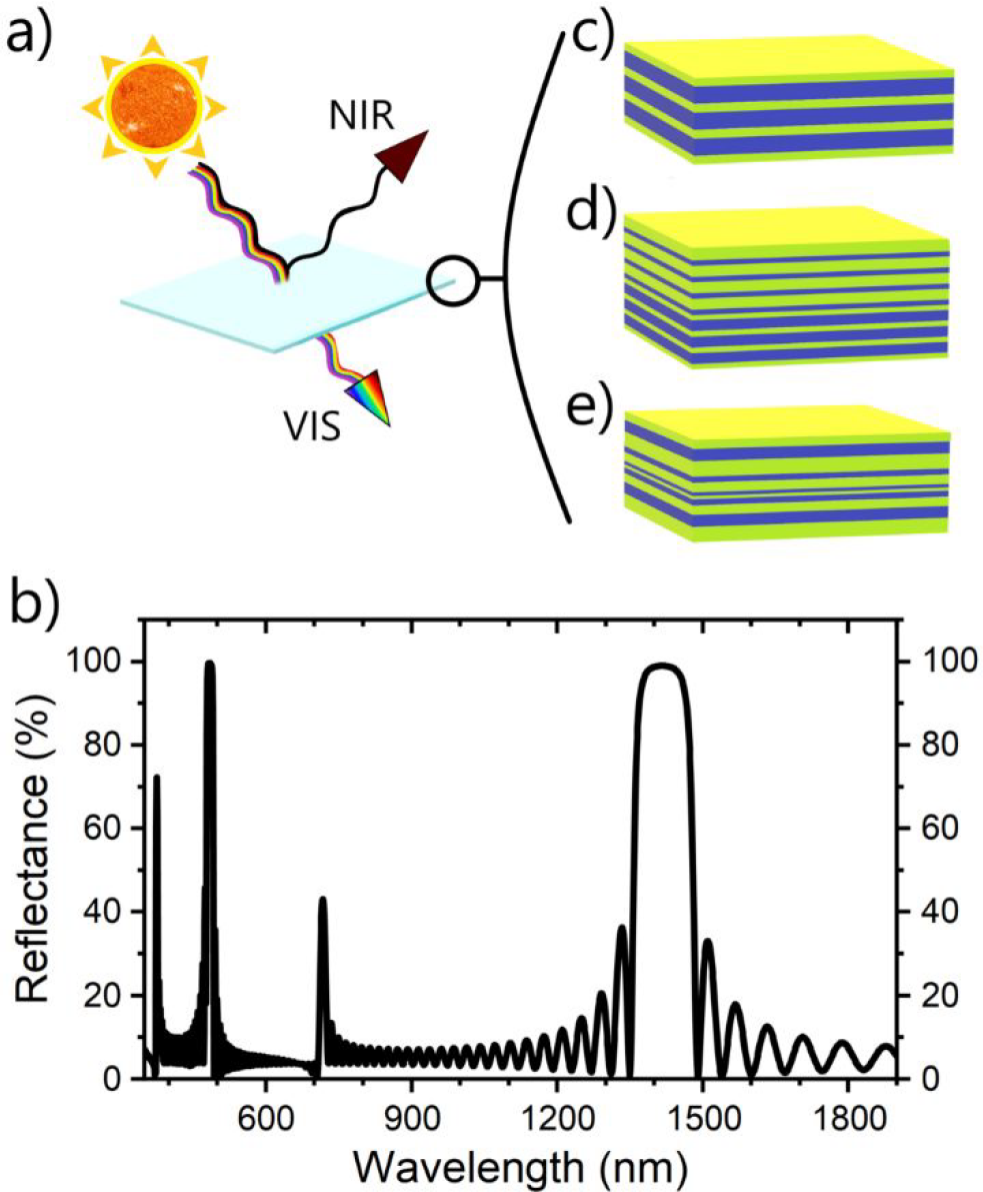
(a) Schematic of aegises functioning, reflecting the NIR sunlight and transmitting the visone. (b) Calculated reflectance spectrum of a typical polymer DBR. (c) Schematic structure of a typical single mirror DBR (aegis, AE). (d) Schematic structure of a tandem DBR formed by two DBRs stacked one on top of the other (tandem aegis, TAE). (e) Schematic structure of a single superperiod in a superperiodic DBR (superperiodic aegis, SPAE).

Given the irradiance of the solar spectrum at ground level and temperate latitudes (Supporting Information, Figure S1),^16^ one can calculate that the energy coming from the Sun is primarily in the visible (vis, 390–780 nm; only region perceived by our eyes) and near-infrared (NIR, 780–2500 nm) ranges, accounting for almost 50% of the total energy each.^15,17−19^ In comparison, the ultraviolet (UV) tail of the spectrum accounts for a few percent, whereas the medium infrared accounts for about 1%. Both UV and NIR light, although invisible to our eyes, can be efficiently absorbed by macroscopic bodies, but the latter contains a greater amount of energy and, even though absorption coefficients are usually larger for the former, this is negligible for macroscopic solids much thicker than extinction length. Thus, we focused particularly on the NIR region. There, the absorption spectra of many molecules show peaks due to overtones and combination bands of vibrational modes;^20^ in correspondence to these peaks, sunlight is absorbed by molecules and converted into heat.^18^ This is the case of −OH and −CH moieties largely present in water and polymers used in packaging, respectively. Thus, using mirrors able to reflect only selected NIR radiation promises to reduce the sunlight heating effect without losing transparency to the eye. To obtain this NIR-reflecting, vis-transmitting effect (schematized in Figure 1a), structures with tunable optical response must be used. Different methods have been proposed in literature, ranging from liquid crystals, whose tunability of optical response is particularly interesting,^21−24^ to delignified wood^19,25^ and assemblies of nano- or microstructures such as wires^26^ or spheres,^27^ and many others.^21,28,29^ While functional, some of these methods tend to lack in selectivity of reflected wavelengths or scalability. Another approach, which we therefore followed in this work, is to exploit all-polymer distributed Bragg reflectors (DBRs), that is, multilayered structures formed by periodically alternating thin polymer films with different refractive index.^29,30^ The periodicity of DBRs, defined by the thicknesses of the repeating layers, has the same order of magnitude of wavelengths of vis-NIR radiation, a few hundreds of nanometers.^31^ In these structures, coherent light diffraction effects conceptually similar to those observed in crystal lattices with X-rays arise. Indeed, the multiple reflections and refractions at the interfaces generate spectral regions in which light rays interfere destructively, impeding light propagation inside the DBR. These regions, called photonic band gaps (PBGs), are detected as high-reflectance regions in the spectrum. Figure 1b displays the calculated reflectance spectrum of a typical polymer DBR (represented with a reduced number of layers in Figure 1c) formed by stacking 25 bilayers of cellulose acetate (CA) and poly(*N*-vinylcarbazole) (PVK). The maximum of reflectance at 1400 nm corresponds to the first order or main PBG. The other maxima, occurring at integer fractions of this wavelength, correspond to higher orders of diffraction. Between PBGs, low-reflectance regions characterized by an oscillating pattern typical of thin-film interference, called interference fringes, can be seen. The spectral position of PBGs can be predicted using the Bragg-Snell law and depends on the thicknesses (*d*_H_, *d*_L_) and refractive indices (*n*_H_, *n*_L_) of the high and low index constituent layers, respectively, and also on the angle by which the light impinges into the crystal.^32,33^ Thus, NIR reflection can be achieved by tuning the constitutive parameters; if the layers are thick enough, the main PBG will be located in the NIR region. PBG’s width increases with the dielectric contrast (Δ*n* = *n*_H_ – *n*_L_) among the materials constituting the DBR, whereas its intensity increases both with Δ*n* and the number of layers.^33,34^ Relative intensities of different PBG orders can be adjusted by varying the ratio between the two layers’ optical thicknesses (*n*_j_·*d*_j_). Conditions such as the quarter wave stack, where the two layers have the same optical thickness (*n*_H_·*d*_H_ = *n*_L_·*d*_L_), can be obtained to maximize the intensity of the even order PBGs and minimize the intensity of the odd ones.^32^

DBRs are usually made of polymer or inorganic materials.^32,33^ While inorganics offer a broad PBG width thanks to the large dielectric contrast provided, the use of polymers as building blocks favors structures with lightweight, mechanical flexibility, and lower costs with respect to the established inorganic counterparts.^29,32,35^ At the laboratory scale, polymer DBRs are made by solution processing such as dip- and spin-coating.^30,32^ Co-extrusion, a technique also used in the packaging industry, has been envisaged to allow structure fabrication on the square meter area.^36,56^ For this reason, polymer DBR structures are arousing increasing interest in different fields including sensing,^37−39^ fluorescence control and lasing,^40−45^ and thermal shielding.^29,30,36^ As mentioned, this comes at the cost of a reduced dielectric contrast typical of mutually processable commercial polymers, thus generating the need to design complex structures or using new materials to increase the PBG width in certain applications.^46−49^

Here, we report on DBRs with highly reflecting PBGs in the NIR spectral range and transparency in the vis one to selectively reflect heating radiations (Figure 1a). A similar approach is used in films to be applied on windows; however, commercial films usually reflect a narrow spectral range of NIR radiation and provide limited transparency.^50^ These structures have been theoretically optimized by Cheng et al., while experimental literature is still quite scarce.^36,51^ The problem was approached in a paper by Nakamura et al.^30^ who obtained good results in thermal shielding, but used polymers charged with inorganic particles to increase the dielectric contrast, and not actual all-polymer structures.^30^ A patent by Radice et al.^29^ instead reported on all-polymer NIR-reflecting DBRs, fabricated by coupling an expensive and hardly processable fluoropolymer (Hyflon) with PVK. This allowed the authors to achieve a high dielectric contrast, and thus wide and intense PBGs.^29^ The DBRs were then tested as thermal shields, reducing the temperature increase of screened objects by about 12% when interposed between them and the light source. In the current research, the latter result is improved, using less expensive and easier to process polymers. The smaller dielectric contrast of these polymers (with respect to Radice et al.^29^) is compensated by an accurate tuning of the DBRs reflectance spectra with respect to the absorbance of the shielded objects, and by using elaborate architectures including tandem and superperiodic ones (Figure 1d,e respectively, described later in Section 3.1). The structures were then tested for the thermal shielding of water and paraffin providing record efficiencies. We call our thermal shields “photonic aegises” (PAEs), referencing the goat pelt worn by the goddess Athena: thin, light, flexible, and modest, nonetheless a formidable protection.^52^

## 2 Experimental Section

### 2.1 Aegises Fabrication

PAEs were created by spin-coating on clear glass substrates sized 25 × 25 × 1 mm^3^, using PVK (Carlo Erba, MW = 135 000) solvated in toluene, alternating it with CA (Aldrich, MW = 50 000) solvated in 4-hydroxy-4-methyl-2-pentanone. For tandem aegis (TAE) TAE2 and superperiodic aegis (SPAE) SPAE1 (see Results and Discussion), poly(acrylic acid) (PAA; Aldrich, MW = 2000) solvated in 4-hydroxy-2-pentanol was used instead of CA. Layers were cast by dynamically spin-coating 100 μL of polymer solutions, alternating between high and low index polymers. The number of layers constituting each structure is reported in Table 1. Concentrations used varied depending on the aegis, remaining in the 40–55 mg mL^–1^ range for PVK, 30–45 mg mL^–1^ for CA, and 60–75 mg mL^–1^ for PAA. Rotation speeds used were also typical of spin-coating, ranging between 80 and 190 revolutions per second.

**Table 1:** Calculated Layer Thicknesses for the Aegises Fabricated (*d*_H_ and *d*_L_), Their Number of Layers (NoL), and Their Calculated Total Thickness (TT)^a^

aegis		*d*_H_ [nm]	*d*_L_ [nm]	NoL	TT [μm]
AE1		141	167	51	7.8
AE2		175	197	51	9.3
AE3		202	259	51	11.6
TAE1	(top)	140	166	40	10.8
	(bot)	178	199	41	
TAE2^b^	(top)	144	158	40	19.8
	(mid)	154	170	40	
	(bot)	161	177	41	
SPAE1^b^^,^^c^		238 to 150 to 238	213 to 151 to 213	30 × 3	16.2
SPAE2		323 to 221 to 323	295 to 200 to 295	30 × 3	22.5

a High refractive index layers (subscript H) are always of PVK, low refractive index ones are of CA unless otherwise noted.

b Low refractive index layer is PAA instead of CA.

c Layer thicknesses in each SPAE’s single superperiod vary with arithmetic progression from maximum to minimum and vice versa. Thicknesses for each layer are reported in Supporting Information Figure S5.

### 2.2 Aegises Characterization

A custom setup based on optical fibers was used to measure the reflectance spectra of the PAEs. Samples were placed under an Avantes BIF-600 UV–vis–NIR optical fiber. The incident light beam was impinged on the PAE surface from DH-2000-BAL (Ocean Optics) deuterium and tungsten–halogen sources (spectral range 230–2500 nm). The reflected signal was relayed to an AvaSpec-ULS4096CL-EVO detector (complementary metal-oxide semiconductor; spectral range 200–1100 nm; resolution 1.3 nm) and to an Arcoptix NIR-Rocket Fourier transform-interferometer (900–2600 nm, resolution 8 cm^–1^) for collection. The reflectance was measured as the ratio between the collected signal and a reference, which is an aluminum mirror.

### 2.3 Spectral Modeling

The modeling of the PAEs reflectance spectra was performed using custom MATLAB code based on the transfer matrix method as reported in previous works,^32,46^ using the polymers’ refractive index dispersions. Fittings of the data allowed calculating the thickness of the constituting layers, reported in Table 1.

### 2.4 Scanning Electron Microscope (SEM) Measurements

Samples were at first frozen in liquid nitrogen and then fractured to reveal the cross section. A thin carbon layer was deposited on the fracture edge by a high vacuum evaporator (Polaron 6700). SEM measurements were then performed using FE-SEM Zeiss SUPRA 40 VP at an acceleration voltage of 5 kV. Images were then analyzed with the software ImageJ, and thicknesses were extrapolated when possible.

### 2.5 Thermal Experiments

To assess the efficiency of PAEs in thermal shielding, different homemade setups were designed and fabricated. The first setup was designed on Autodesk’s Fusion 360 and then 3D-printed out of polylactic acid (PLA); it is pictured in the Supporting Information Figure S2a. It allowed measuring the temperature of a piece of Parafilm, about 25 × 25 mm^2^, while the light of an illuminator (Edmund Optics Model 21AC), carried by the associated polymer optical fiber, was impinged onto it. The fiber head (not shown in Figure S2a) was inserted into the relative holder. A slot for the insertion of an aegis or a clear reference glass was present between the light source and the sample. After switching the light on, the temperature was measured over time with a flat probe Testo 110 digital thermometer (range, −50/150 °C; resolution, 0.1 °C). Every measurement using an aegis was followed by a reference one; then, data were normalized and averaged.

For water measurements a PC-linkable, dual channel thermocouple thermometer TSP-01 from Thorlabs (range −20/110 °C, resolution 0.05 °C) with two immersion probes was used instead of Testo. This way, measurements were performed at the same time for the reference and aegis. Controlled volumes of deionized water in clear glass vials with a diameter of 23 mm were used as samples. Two different custom-made setups were made for water measurements; in one setup, the light source used was a powerful incandescent lamp (Philips IR250, 250 W), and in the other a group of LEDs was used. The front side of the first setup, which faces the lamp, is represented in Figure S2b. A removable piece allowed for two aegises and two references to be used simultaneously to cover the windows cut through a black screen, where light can pass through. An additional screen covered in aluminum foil (bottom left of the panel) was placed in front of the black screen during the measurements. The vials were placed behind the windows, as represented in Figure S2c, and their contents’ temperature was measured over time. Because of the asymmetry of the lamp’s emission, the measurements were performed two times for each pair of aegises, exchanging positions with the references each time.

The second setup, also 3D printed, was fitted with a homemade circuit and it is represented in Figure S2d (interior) and Figure S2e (exterior). The circuit powered with a current of 59 mA two groups of four LEDs each (Thorlabs 5 mW 970 nm). These provided illumination to each of the two water vials through the aegis/reference, as visible in Figure S2e. The main body of the TSP-01 thermometer was used to measure ambient temperature near the vials, whereas a 12 V typical PC vent was mounted atop of the setup to cool down the LEDs, avoiding their heating up.

## 3 Results and Discussion

### 3.1 Aegises Rationale and Design

As most substances absorb NIR radiation at different and specific wavelengths due to overtones and combination bands of vibrational modes, efficient shielding requires a precise tuning of the optical response of the PAEs. In principle, this can be achieved by engineering the thickness and refractive index of the PAEs building blocks. However, in a real-life scenario, sunlight impinges on surfaces with different angles and different and/or combined substances may need to be shielded. Since the PBG spectral position shifts toward smaller wavelengths as light’s incidence angle increases (see Section 3.2),^32^ an absorption peak perfectly screened at normal incidence could lose correspondence above certain angles, reducing the shielding efficiency. Furthermore, as just mentioned, each substance shows different absorption peaks, thus requiring high reflectance in many different spectral regions, complicating the engineering of the structure. Both issues can be solved via engineering wide PBGs, either by using complex tandem structures or by increasing the dielectric contrast of materials composing the structure. We tested a series of PAEs made by PVK (*n* = 1.68 at 600 nm)^32^ and either CA (*n* = 1.46 at 600 nm)^42^ or PAA (*n* = 1.51 at 600 nm).^46^ These materials allowed obtaining a relatively high dielectric contrast (0.22 and 0.16 respectively), while keeping a good processability and thus ease of fabrication of complex structures. The PBG width was then maximized by designing three types of aegises: (i) single DBR AEs (Figure 1c), (ii) TAEs (Figure 1d) and (iii) SPAEs (Figure 1e). AEs are well-known in literature for sensing,^37^ fluorescence control,^40−45^ and thermal shielding applications,^29,33,36^ and are formed by repeating a single period, and TAEs are formed by stacking AEs tuned to different wavelengths one on top of the other.^32,36^ We also tested the effect of the quarter-wave condition (all layers of equal optical thickness) to maximize the main PBG’s width and remove even-ordered PBGs, thus obtaining visible transparency. SPAEs, instead, are fabricated repeating three times a single superperiod in which the bilayers’ thicknesses gradually decrease and then increase again (Figure 1e). This is a variation of the wide-PBG inorganic structures with gradually increasing layers’ thicknesses^34,53^ to compensate the lower dielectric contrast achievable with polymers.

### 3.2 Aegises Optical Characterization and Modeling

**Figure 2:**
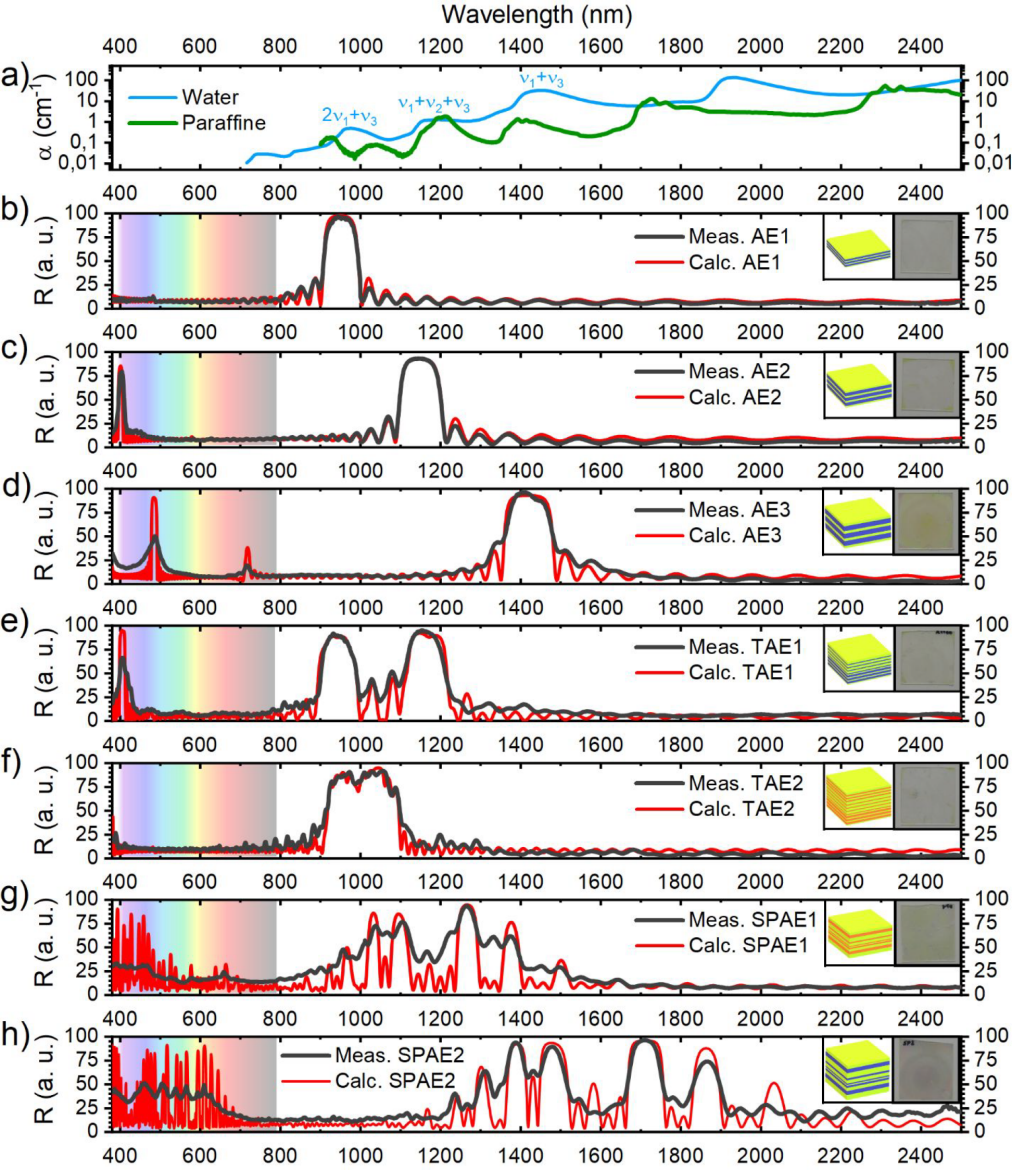
(a) Water (blue) and paraffin (green) absorbance spectra. Water absorption peaks due to overtones and combination bands are labeled as sum of fundamental vibrational modes (symmetric stretching *v*_1_, bending *v*_2_, and asymmetric stretching *v*_3_). Measured (black lines) and calculated (red lines) reflectance spectra of aegises (b) AE1, (c) AE2, (d) AE3, (e) TAE1, (f) TAE2, (g) SPAE1, and (h) SPAE2. In the insets, a schematic (left) and a top-view picture on a white background (right) for each sample.

Figure 2 compares and lines up the spectra of the two prototype materials to be shielded (panel a), with the PAEs’ reflectance spectra (panels b-h). The chosen media were water and paraffin. The spectrum of water shows distinctive peaks at 950, 1200, 1450, and 1950 nm due to overtones and combination bands of the three fundamental vibrational modes (symmetric O–H stretching *v*_1_, H–O–H bending *v*_2_, and asymmetric O–H stretching *v*_3_).^54^ The spectrum of paraffin instead shows several peaks arising from combinations of −CH, −CH_2_, −CH_3_, and −OH groups vibrational modes at 900, 1050, 1200, 1400, 1800, and 2200 nm. Notice that we report the data for liquid paraffin to better highlight absorbance in the low-wavelength range, while the shielding experiment was performed on a solid Parafilm sample. This is due to the presence of scattering in the shorter wavelength range for the solid film that does not allow properly resolving certain peaks. However, the two spectra are almost identical, as reported in Supporting Information Figure S4.

To shield these materials, seven PAEs were fabricated. Three of them were AEs, showing PBGs at 950 nm (AE1, Figure 2b), 1160 nm (AE2, Figure 2c), and 1430 nm (AE3, Figure 2d). AE1 and AE2 are transparent and colorless in the visible part of the spectrum, whereas AE3 looks slightly yellow in transmission. Its spectrum in Figure 2d shows the main PBG at 1430 nm, whereas the partially suppressed second and third order can be seen around 700 and 450 nm, respectively, and give AE3 its color. A picture of an identical PAE, detached from the glass substrate, is reported in Supporting Information Figure S3 to show the flexibility of the films and their resistance to the free-standing condition. The three AEs are tuned to shield a corresponding absorption peak of liquid water and paraffin. Another two were TAEs made of a double and triple mirror, respectively. The first (TAE1, Figure 2e) shows PBGs at 950 and 1150 nm, and it is effectively schematized by stacking AE1 and AE2. The PBGs correspond to the two absorption peaks of water and of paraffin. Between the PBGs, two intense interference fringes allow good reflectance in an extended region. No second order PBGs are observed, certifying the structure as a tandem of two quarter-wave DBRs (i. e., with layers of equal optical thickness). The third order relative to the 1150 nm PBG is observed at about 400 nm, but eye sensitivity is very low at those wavelengths and thus TAE1 looks transparent.^55^ The second TAE is a triple mirror built by placing in tandem three-quarter-wave, transparent, and colorless DBRs. It shows a 200 nm wide, structured PBG centered at 970 nm (TAE2, Figure 2f), which corresponds to the partial superimposition of the main PBG and interference fringes of each constituting DBR. Second order PBGs are canceled, as per quarter-wave condition, whereas in the UV region a third-order peak can be seen at around 380 nm. As described later, TAE2 was meant to reflect most of the light emitted by a 970 nm LED used as a light source. Two superperiodic structures instead were made to show multiple PBGs, the first sample in the 900–1300 nm region (SPAE1, Figure 2g), the other one in the 1300–1900 nm region (SPAE2, Figure 2h). The spectrum of SPAE1 in Figure 2g is a typical SPAE spectrum with a high-reflectance region formed by a series of close peaks between 950 and 1400 nm with maxima around 1100 and 1300 nm. In the vis region, a plateau around 450 nm and some low-intensity peaks make the structure appear colorless with a total transmittance around 75%. The spectrum of SPAE2 in Figure 2h shows the main reflectance region formed by six main peaks around 1250, 1300, 1400, 1500, 1700, and 1900 nm with an overall high reflectance in the NIR region. In the vis range, SPAE2 shows a reflectance plateau in the blue-yellow region.

The optical responses of our PAEs (black lines in Figure 2b–h) were also fitted via transfer matrix method calculations (red lines in the same figure) to obtain the most probable thicknesses of the repeating layers, as described in previous works.^32^ Good agreement between measured and calculated spectra was obtained for all samples, especially concerning the main PBGs in the NIR region. Minor differences are detected at shorter wavelengths where light scattering phenomena may affect the experimental data. The thicknesses obtained by fitting the calculated spectra to the data are reported in Table 1.

**Figure 3:**
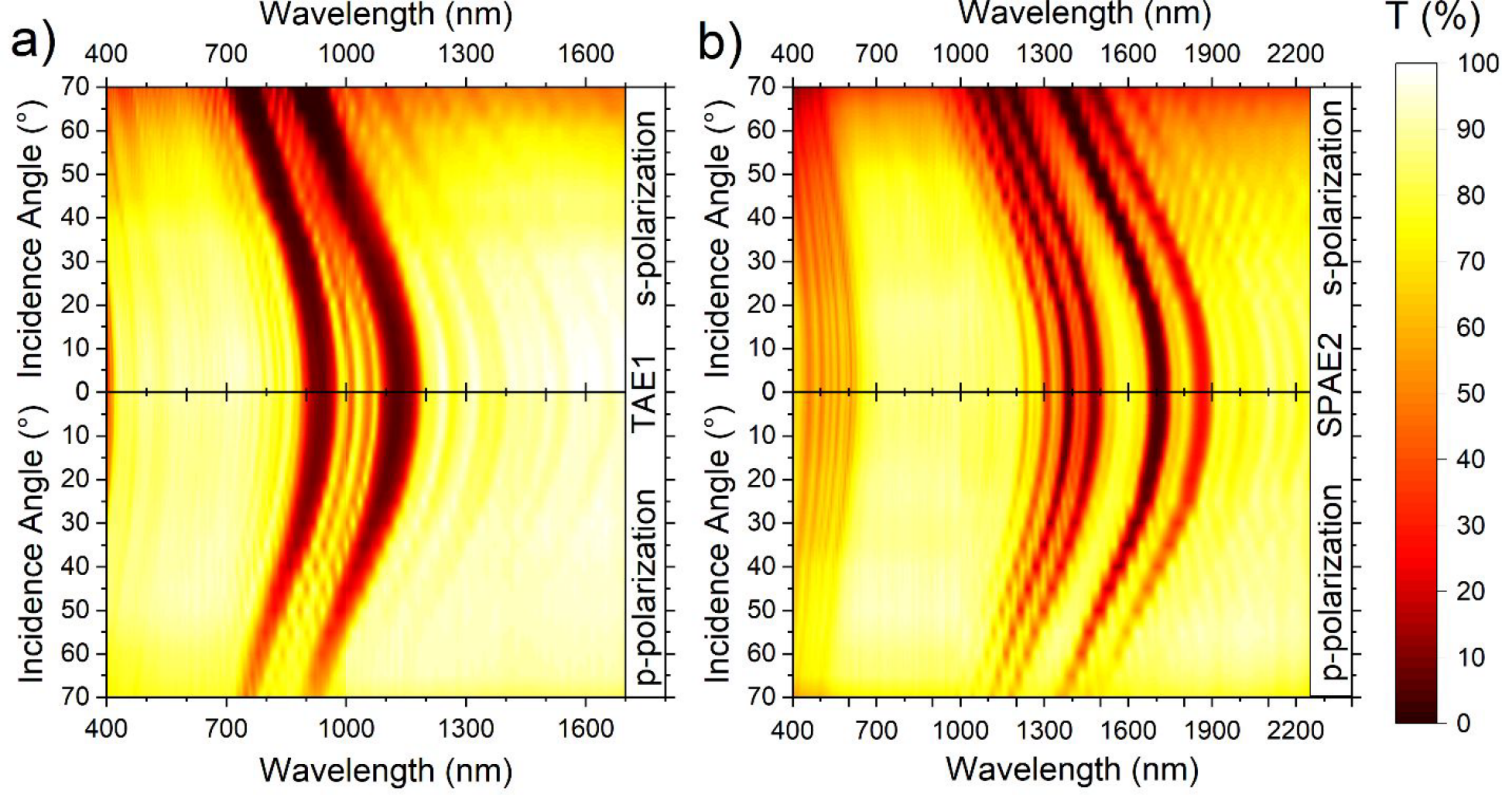
(a) Transmission spectra of TAE1 for angles 0–70° every 5°, in perpendicular (top) and parallel (bottom) polarization. (b) Transmission spectra of SPAE2 for angles 0–70° every 5°, in perpendicular (top) and parallel (bottom) polarization.

As stated in Section 3.1 and well-known from literature, DBRs show angular dispersion. Indeed, the position of the PBG shifts toward shorter wavelengths when increasing the angle of incidence of light with a different behavior dependent on light polarization.^31,32,37^ This can be important in thermal shielding applications since sunlight impinges on surfaces at different angles during the day. Therefore, Figure 3 reports the angle-resolved transmittance spectra for TAE1 (panel a) and SPAE2 (panel b) to show aegises’ typical angular dispersion.

Figure 3a shows that increasing the angle of incidence for s-polarized light (top plot) TAE1’s PBGs shift toward smaller wavelengths and widen, while the overall transmittance decreases. In p polarization (bottom) PBGs become narrower by increasing the incidence angle, and the overall transmittance increases until Brewster’s angle is reached (around 60°). After Brewster’s angle, the overall transmittance decreases. The behavior, for both polarizations, is the same for TAE1 and SPAE2, too.

### 3.3 SEM Image Analysis

**Figure 4:**
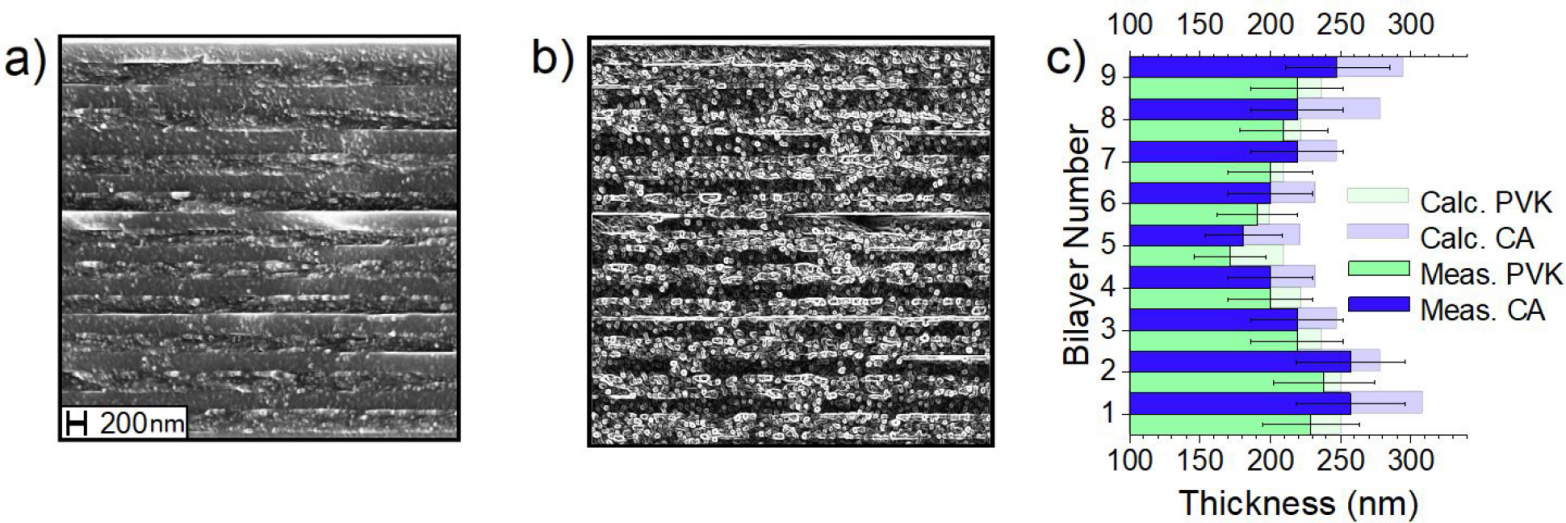
(a) SEM image of the cross-section of SPAE2. (b) Same picture after the contrast enhancement. (c) Measured (solid bars) and calculated (faded bars) for sample SPAE2.

To further validate the calculated thicknesses, we performed scanning electron microscopy analysis on two chosen samples, TAE1 and SPAE2, as they are the two most complex structures. Both samples delaminated heavily during sample preparation for SEM imaging due to the freeze-cracking cut; the only visible part of SPAE2, consisting in 20 layers, is reported in Figure 4a. The layers can be distinguished, although the interfaces between them are blurred; PVK layers are the most compact ones in which the globular structures are less visible. Layer thicknesses were extrapolated from this picture after enhancing the contrast (Figure 4b) but due to the difficulty in identifying the interfaces and the limited resolution of the picture, a considerable error persists in the measurements, which is intrinsic to sample preparation.^57^ This is shown in Figure 4c, which reports the thicknesses measured for each bilayer as bright bars, discriminating between PVK (cyan) and CA (blue). The derived values from optical modeling are reported in the same figure as faded bars. Considering the errors, the agreement is quite good, as the process of preparing and measuring the sample is the cause for the discrepancies. The thicknesses are compatible with the calculated values, and the trend of the thickness variation is the expected one for the superperiodic structures, confirming the validity of the transfer matrix results. Some additional comments and the pictures of TAE1 are reported in Supporting Information Section S6.

### 3.4 Thermal Shielding Efficiency

The PAEs shielding efficiency was assessed by interposing alternatively an aegis or a reference between a light source and a sample while measuring and comparing its temperature increase (see Experimental Section for details). A simplified model for heating, reported in Supporting Information Section S7, was developed to obtain the essential parameters of the process. The model describes an asymptotic growth for the temperature increase over time, reported in eq 1
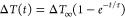
1

The terms equilibrium temperature (*ΔT*_*∞*_,) and the characteristic time of the process (τ) are found to be dependent on the incoming power, *w*_lamp_, the mass and specific heat of the body (*m* and *c*, respectively), and the heat exchange factor *h*, which is characteristic of the setup geometry and the subsequent heat exchange
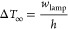
2a
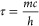
2b

Equation 1 with eqs 2a and 2b predicts the temperature increase over time in heated samples and will therefore be used to fit the experimental data and extrapolate the *ΔT*_*∞*_ and τ parameters.

#### 3.4.1 Shielding Experiment: Parafilm

**Figure 5:**
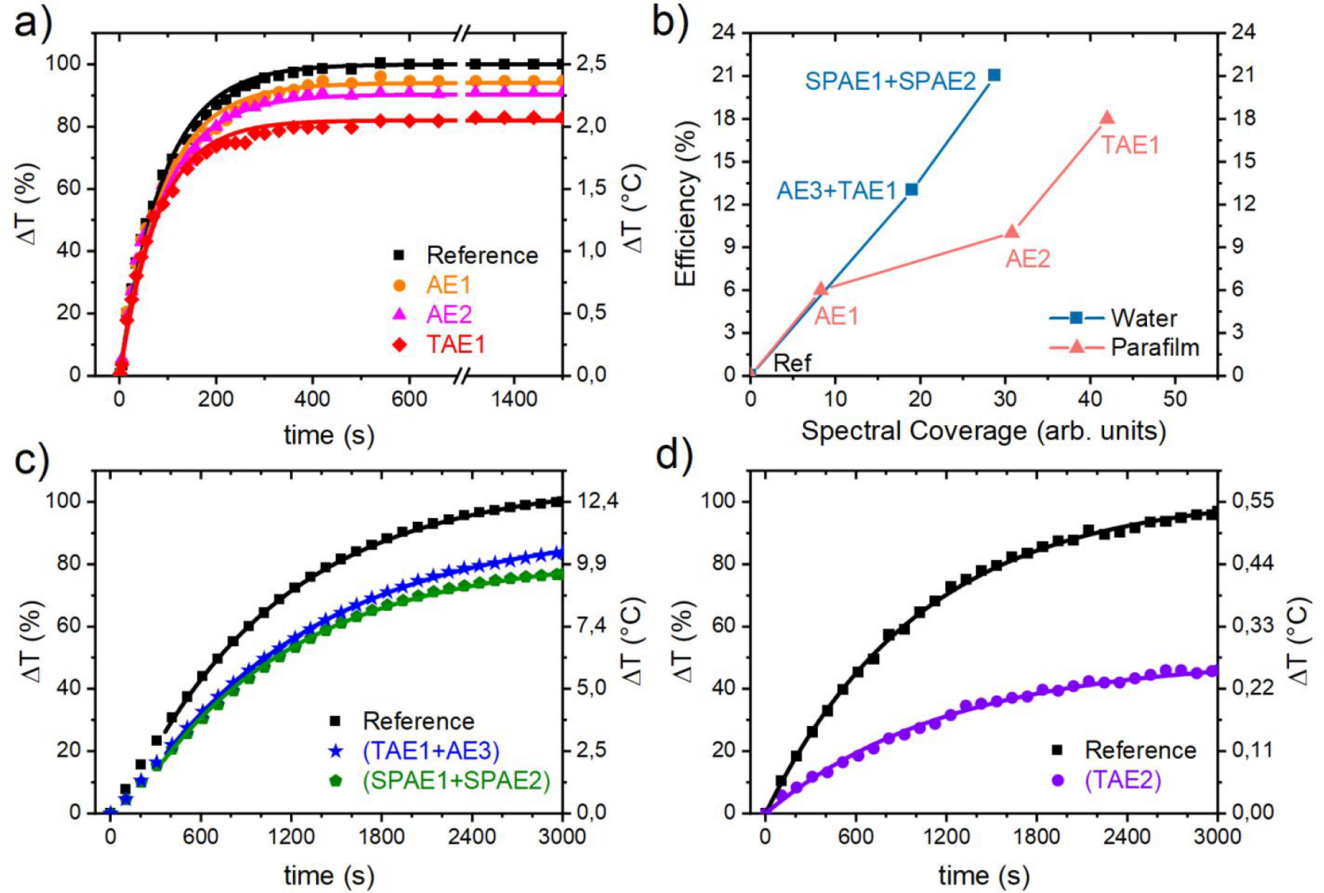
Temperature increase, measured over time during thermal experiments, for (a) Parafilm sample, (c) water sample illuminated with incandescent bulb, and (d) water sample illuminated by 970 nm LED. Marks are experimental data; continuous lines are fittings. (b) Efficiency of the aegises and reference against the respective spectral coverage values. Lines are only a guide to the eye.

The first shielding experiment was run on a solid paraffin film (Parafilm). Figure 5a shows that the Parafilm dynamic temperature increases upon irradiation when shielded by a reference of clear glass substrate or different aegises cast on the same type of substrate: AE1 (orange circles), AE2 (magenta triangles), or TAE1 (red rhombuses). The trend is the same in the four cases, that is, a linear rise in temperature followed by a plateau assessing to the equilibrium value. The lowest *ΔT*_*∞*_ was obtained for TAE1, followed by AE2 and AE1. The curves were then fit with eq 1 to extrapolate the *ΔT*_*∞*_ equilibrium term reached by using each aegis. *ΔT*_*∞*_ terms were thus used to obtain an efficiency value for the aegises, defined as η = 1 – Δ*T*_∞,aegis_/Δ*T*_∞,ref_. The fittings converged easily toward a good agreement between data and model. The obtained efficiencies are 6% for AE1, 10% for AE2, and 18% for TAE1 (Table 2). This result can be explained observing the liquid paraffin absorption spectrum (comparable to the one of the solid film in Supporting Information Figure S4) in Figure 2a. Aegis AE1 (Figure 2b) shows a PBG centered at 950 nm which partially superimposes the paraffin absorption peak centered at 900 nm. AE2 (Figure 2c) shows a PBG at 1160 nm, partially overlapped to the absorbance peak at 1200 nm, which is more intense with respect to the former, explaining the higher efficiency of AE2. Along of showing both peaks, sample TAE1 shows intense fringes in the spectral region between the two PBGs, partially screening the 1000 nm absorption peak of paraffin. The higher efficiency is due to this simultaneous screening of the three peaks.

**Table 2:** Values of Equilibrium Temperature (*ΔT*_*∞*_), Shielding Efficiency (η), Characteristic Time (τ), Delay (*t*_0_), and Heat Exchange Coefficient (*h*) Obtained for Reference and Aegises by Fitting the Plots in Figure 5a,c,d

aegis	material	*ΔT*_*∞*_ [°C]	η [%]	τ [s]	*t*_0_ [s]	*h* [mW K^–1^]
reference	Parafilm	2.50	0	80		1.43^a^
AE1	Parafilm	2.34	6	73		1.57^a^
AE2	Parafilm	2.27	10	77		1.49^a^
TAE1	Parafilm	2.05	18	74		1.56^a^
reference	water	13.0	0	1140	80	30^b^
TAE1+AE3	water	11.5	13	1180	116	28^b^
SPAE1+SPAE2	water	10.6	21	1210	118	28^b^
reference	water^c^	0.55	0	∼1100		27^b^
TAE2	water^c^	0.26	∼52	∼1100		27^b^

a The heat exchange coefficient *h* is calculated from τ according to its definition (eq 2), known Parafilm mass to be 0.05 g and assuming specific heat 2.3 J g^–1^ K^–1^.^58^

b *h* is calculated as stated before, known water mass to be 8 g and its specific heat 4.196 J g^–1^ K^–1^.

c Light source is the LED.

To express quantitatively these differences in efficiency, we defined a parameter, called spectral coverage (*S*). *T*_PAE_(λ), transmission spectrum of an aegis, *T*_ref_(λ), transmission spectrum of the reference, and *A*(λ), absorbance spectrum of the material need to be shielded (in Figure 2a)
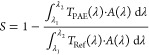
3

The integration was numerically performed; in the Parafilm case, by integrating between 900 and 1400 nm that is the spectral range in which all PBGs are located. The efficiencies for the various PAEs are thus reported against the respective spectral coverage values obtained with eq 3, as pink triangles in Figure 5b. It can be seen that by increasing the spectral coverage, the efficiency increases; as expected, spectral coverage depends on both the tuning of PAEs to the peaks they shield and the absorption intensity of the shielded peak.

These results hold value on two different levels. First, the shielded absorption peaks of the paraffin film are assigned to overtones and combination bands of C–H bonds.^18^ Such transitions with slight variations are typical of most organic substances and polymers.^18^ Thus, here it is shown how a vis-transparent, NIR-reflecting aegis can reduce the temperature increase in an entire class of materials ubiquitously used, for example, in packaging. Compared to the result reported by Radice et al.,^29^ which corresponds to an efficiency of 12%, these results represent an improvement on three different fronts: the better performances, the use of materials with higher processability, and the design of the structure which allows obtaining such results with polymers bearing relatively low dielectric contrast (0.22 for this work against 0.34 for Radice et al., see Section 2).^29^

#### 3.4.2 Shielding Experiments: Water, Incandescent Lamp

We employed two different approaches to observe the efficiency of the aegises in different spectral coverage conditions. In the first one, the light source was a powerful incandescent bulb, thus a broadband source, whereas in the second one, 970 nm LEDs were used as narrow-spectrum emitters. In the first case, because of the larger amount of material to shield with respect to the previous case (8 g of water against 0.05 g of Parafilm), we employed a more powerful light source (see Experimental Section). To make the shielding effect more clearly observable, we used two aegises at a time to screen the sample. The pairs used were TAE1+AE3 and SPAE1+SPAE2. The former pair was chosen as a selective reflector for light in close spectral proximity to the first three absorption peaks of water and as a structure generally well-known in literature. The latter acts as a broadband reflector with greater total spectral coverage, and it is used to test the efficacy of the novel superperiodic structures presented. The time to reach the equilibrium temperature was longer in this case due to the larger mass and specific heat of water that both increase τ (eq 2b). The measured heating curves, reported in Figure 5c, present a delay time (*t*_0_, see Supporting Information, Section S8) so that eq 1 was modified
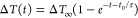
4

The fitting with eq 4 allowed us to retrieve Δ*T*_∞_ and the efficiency of the aegises that are 13% for the pair TAE1+AE3 (respectively the tandem aegis with peaks at 950 and 1160 nm, and the aegis with a 1450 nm peak), and 21% for the pair SPAE1+SPAE2, (the two superperiodic aegises), as reported in Table 2. We calculated the spectral coverage (as per eq 3) for the two pairs, integrating between 780 and 2400 nm. The pairs’ efficiencies were therefore reported against the respective spectral coverage values as blue squares in Figure 5b. As expected, the calculated spectral coverage is greater for SPAEs, whereas it is lower for the TAE1+AE3 pair, as is for their efficiency. This result arises from the broader reflectance of the SPAEs in the NIR region, their larger reflectance intensity with respect to the other sample, and from the presence of scattering from defects in the sample SPAE2. Note that the two sets of data reported in Figure 5b are not meant to be compared with each other, since the efficiencies were measured in different situations and the spectral coverage calculated over different ranges.

#### 3.4.3 Shielding Experiment: Water, LED

To assess the theoretical efficiency limit, we used a second approach of irradiating liquid water with a source whose wavelength corresponds perfectly to one of the absorption peaks (Figure 2a). For this reason, an array of 970 nm LEDs was employed. This test mimics the situation where every absorption peak is shielded when using white light. Intuitively, the *ΔT*_*∞*_ was much smaller with respect to the other experiments due to the low power of the LEDs so that 100% corresponds to about 0.55 °C. Figure 5d shows the temperature increases of the water when shielded by TAE2 or its reference. This, alongside the long time taken by the measurements, forced us to consider the room-temperature variations (in the previous cases, negligible with respect to *ΔT*_*∞*_). Thus, the dynamic room-temperature variations were subtracted from the measured curves to filter out environmental influence. The data were then fitted with eq 1 to retrieve the data reported in Table 2. The efficiency reaches η = 52%, which represents the maximum values achievable with the PAEs when all of the peaks are shielded. This certifies the possibility for transparent aegises to shield substances with high efficiencies in the presence of a good spectral coverage of absorptions, therefore opening perspective for novel processable materials providing larger dielectric contrast recently reported in literature.^46,47^ These materials could in principle favor wider, more intense, and selective PBGs able to shield absorption peaks even when the angle of incidence is different from the normal.

## 4 Conclusion

This work demonstrates that polymer dielectric aegises can efficiently shield materials against undesired radiative heating. Different spin-cast multilayered structures made of commercial polymers commonly used in the field reduced radiative heating from various light sources up to 20% with maximum efficiency reaching 52%. The observed reduction strongly depends on the incident spectrum, the amount of light reflected by the PAEs, and the agreement between the spectral position between the photonic band gap and the absorption peaks of the shielded material (spectral coverage). This efficiency is indeed boosted by tuning the PBG spectral position to where the absorption coefficient of the material to be shielded is larger (spectral coverage), while using the same materials and structure type, or by increasing the complexity of structures and thus the overall reflectance. Superperiodic structures showing a wide, multipeak PBG display indeed the highest efficiency even though this came at the cost of lower transparency in the vis region. Thus, the results obtained are promising in the field of thermal shielding.

## Supporting Information Available

Eight sections, labeled S1–S8, contain the solar spectrum (S1), pictures of the setups for the thermal experiments (S2), a comment and a picture on aegises flexibility (S3), the comparison of paraffin and Parafilm absorption spectra (S4), the calculated thicknesses of SPAE1 and SPAE2 (S5), the SEM measurements of TAE1 and additional comments on the subject (S6), the model for temperature increase in irradiated samples and the derivation thereof (S7), and additional details on water thermal shielding measurements (S8) (PDF)

## ABBREVIATIONS

AE: aegis
CA: cellulose acetate
DBR: distributed Bragg reflector
NIR: near-infrared
PAA: poly(acrylic acid)
PAE: photonic aegis
PBG: photonic band gap
PVK: poly(*N*-vinylcarbazole)
SPAE: superperiodic aegis
TAE: tandem aegis
UV: ultraviolet
vis: visible
