# Supplementary material for: Multilayer Polymer Photonic Aegises Against Near-Infrared Solar Irradiation Heating
Source: ACS Appl Mater Interfaces. 2022 Mar 19;14(12):14550–60. doi: 10.1021/acsami.1c25037 (PMC8972252; doi:10.1021/acsami.1c25037)
Supplement: Supplementary file 1 — am1c25037_si_001.pdf [file am1c25037_si_001.pdf]

SUPPORTING INFORMATION

Multilayer Polymer Photonic Aegises Against Near-Infrared Solar Irradiation Heating

*Andrea Lanfranchi, Heba Megahd, Paola Lova, and Davide Comoretto**

Università degli Studi di Genova - Dipartimento di Chimica e Chimica Industriale, Via Dodecaneso 31, 16146, Genoa, Italy

*E-mail: davide.comoretto@unige.it

S1. AM 1.5 SPECTRUM

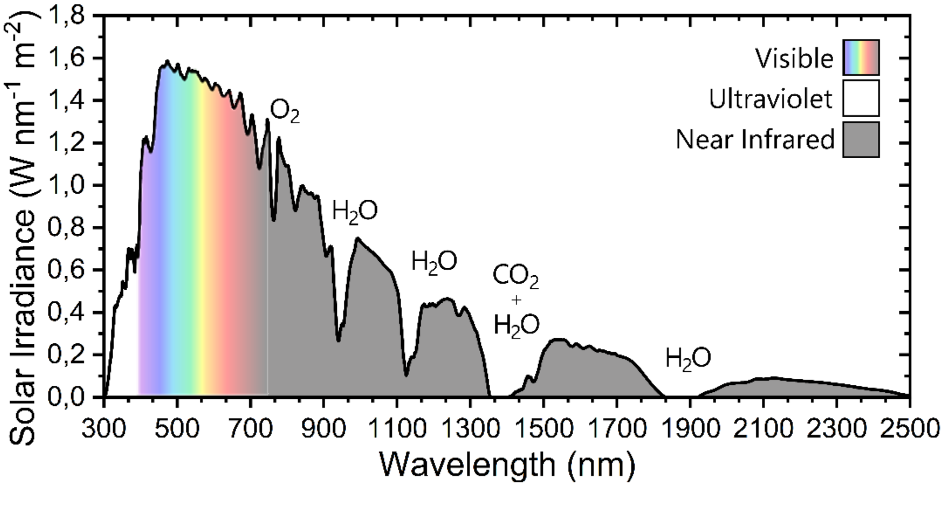

Figure S1. Solar spectrum on Earth's surface. Labels identify the molecules responsible for the irradiance minima.

Figure S1 reports the irradiance of the solar spectrum on earth's surface, at temperate latitude.[1] The visible region is colored, whereas the ultraviolet is white and the near-infrared is grey. The different atmospheric absorption peaks, here observed as minima in irradiance, are labeled with the responsible absorbing molecule(s).

S2. PICTURES OF HEATING SETUPS

The pictures of the setups used are reported here as described in the main text.

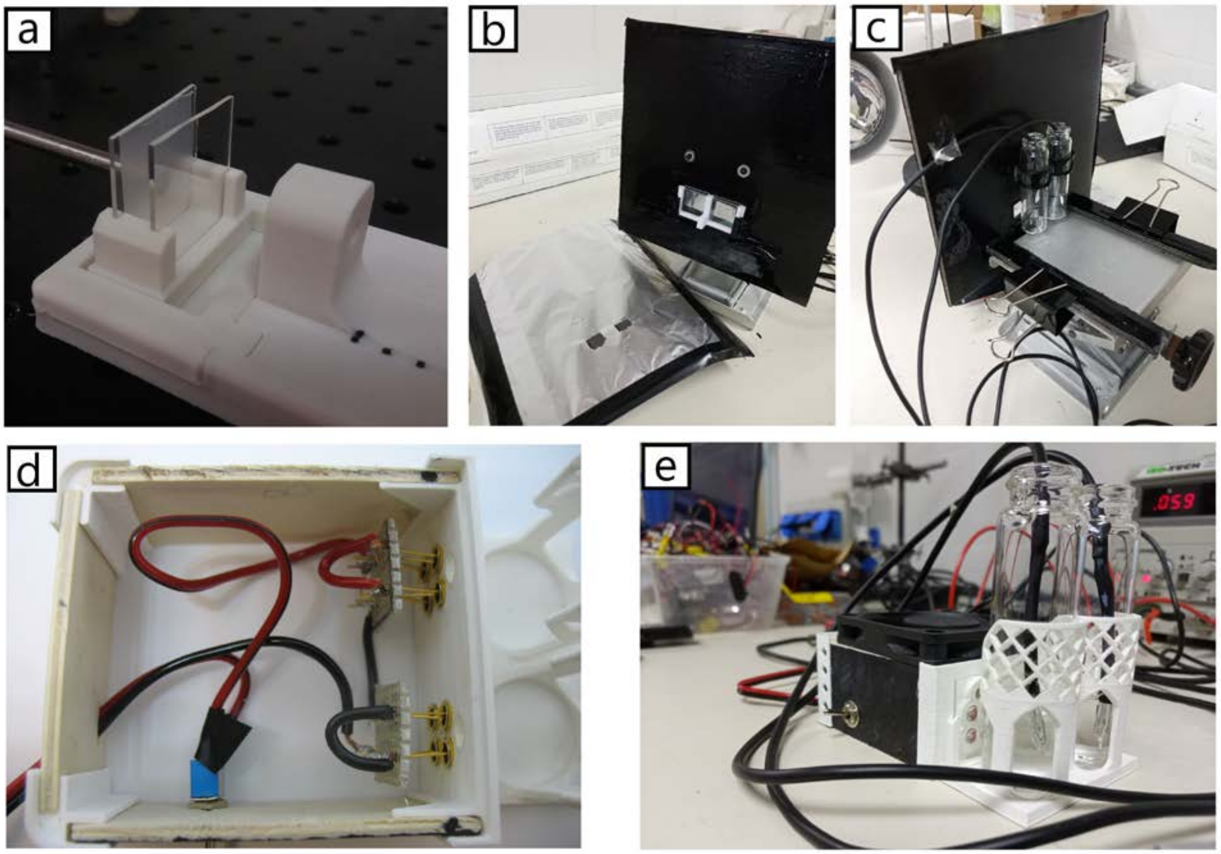

Figure S2. **a)** Setup for the Parafilm™ thermal experiments. **b,c)** front and back respectively of the setup for the water thermal experiments using an incandescent lamp as a light source. **d,e)** interior and exterior respectively of the setup for water thermal experiments with LED light source.

S3. FREE-STANDING AEGIS

It has been already demonstrated in literature that full polymer, poly(*N*-vinylcarbazole)-based dielectric mirrors can be spin-coated onto flexible substrates.[2,3] The results were functional photonic structures that retained a degree of flexibility, meaning that the dielectric mirror itself is flexible. However, the possibility of having free-standing full polymer DBRs is often impeded by the low overall thickness, which may result in a fragile film. The aegises, instead, are constituted by a high number of relatively thick layers, thus they result sturdier. Figure S3 reports a picture of an aegis equivalent to AE3 (recognizable by the slight blue reflections), which was peeled off its glass substrate. The sample is held from one of its corners and tends to roll itself up, demonstrating both its flexibility and its possibility to exist free-standing without breaking.

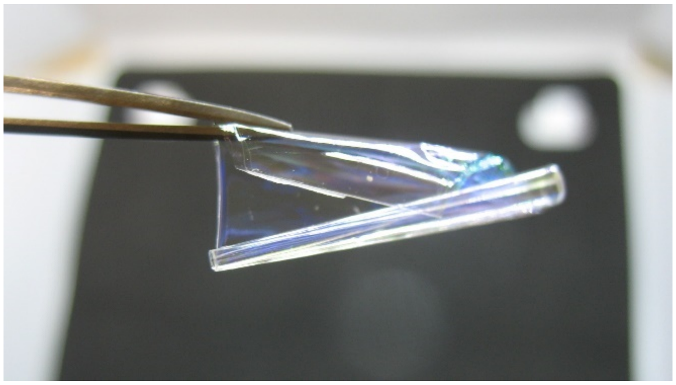

Figure S3. Picture of a free-standing aegis equivalent to AE3, peeled off the glass substrate.

S4. PARAFILM™ AND PARAFFINE MEASUREMENTS

The absorption spectrum of Parafilm™ is very similar to the paraffine one, as reported in Figure S4. Apart from the noisier measurement overall in the Parafilm™ case due to scattering, which makes it impossible to distinguish fine features and low-absorption regions, the agreement is very good.

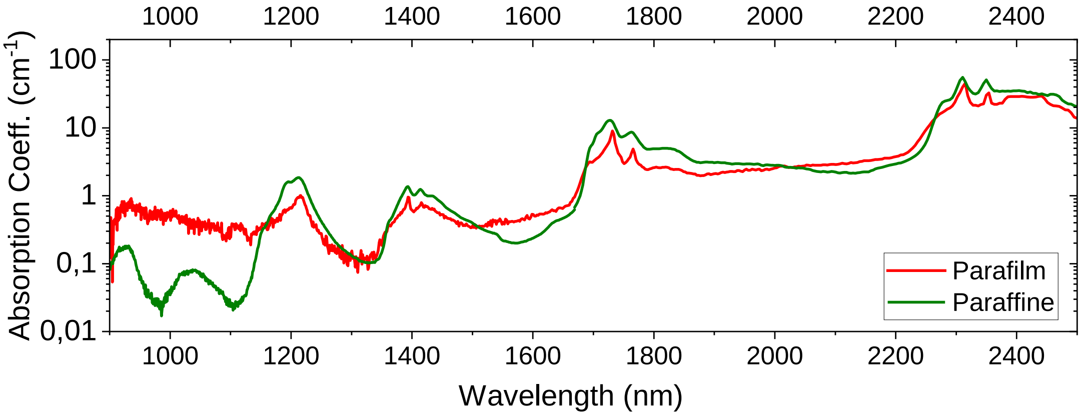

Figure S4. Absorption spectra of Parafilm™ (red line) and liquid paraffine (green line).

S5. CALCULATED THICKNESSES OF SUPERPERIODIC AEGISES

To adequately represent the calculated thicknesses of SPAEs' layers, two bar plots are reported in Figure S5. They represent respectively SPAE1 and SPAE2. Each layer's thickness is indicated by the length of the bar, whereas its position inside the crystal is given by the bilayer number.

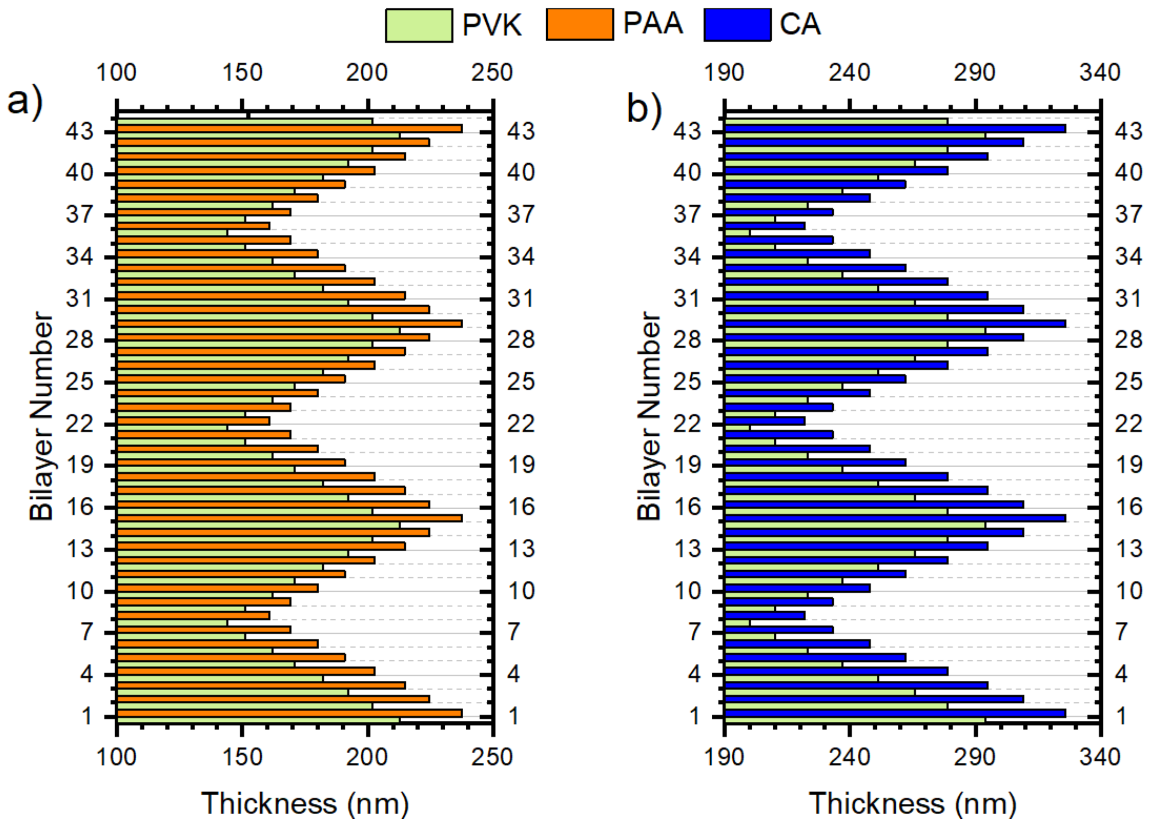

Figure S5. Calculated layer thickness pattern for **a)** SPAE1 and **b)** SPAE2.

S6. SEM ANALYSIS OF AEGISES

As described in the main text, both samples heavily delaminated due to the freeze-cracking process; layers detached from each other in multiple points. As far as SPAE2 is concerned, a relatively thick piece formed by around 20 layers was clearly observable. An uncropped version of Figure 4a is reported in Figure S6a, whereas a magnification of it is represented in Figure S6b.

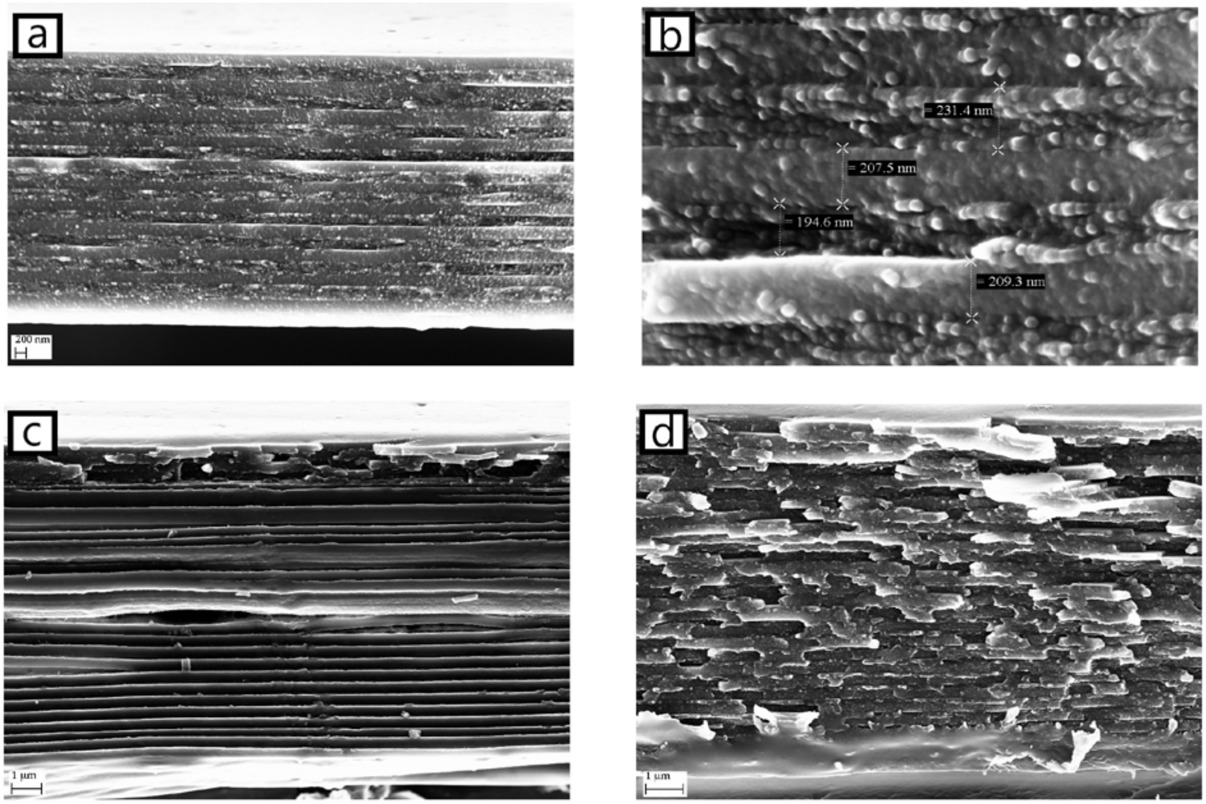

Figure S6. SEM images of **a)** Fractured edge of SPAE2, **b)** Magnification of a small portion of the fractured edge of SPAE2, **c)** and **d)** different portions of the fractured edge of TAE1.

Even though the pictures were useful to obtain thickness values, as reported in the text, many issues negatively affect these measurements. i) it is difficult to obtain a complete cross-section in these multilayer polymer structures, thus leaving the interfaces blurred. ii) the graphitization process, essential to measure the samples, can enhance the defects in the fracture surface. iii)

being thin polymer sheets, the samples heat up quickly when the electron beam is impinged onto them. Such effects lead to drifting in the pictures, an effect that causes focusing problems and limits the scan speed to faster values, contributing to the blurring.

In Figure S6 c-d two different portions of TAE2's fracture edge are represented. The sample delaminated around its middle point and fractured unevenly, as shown by the figure. The fracture is very different in the two pictures, and nonuniform in both of them. Due to the latter problem, it was impossible to obtain accurate results. A selective analysis was performed in some of the best spots and suggested the correct order of magnitude for the thicknesses (100 - 200 nm), simultaneously confirming that PVK layers are thinner than CA ones.

S7. HEATING MODEL

The situation considered is schematized in Figure S7. A body, initially in equilibrium with the surrounding environment at T_{env}, receives via an external source a power w_{lamp}, heating up. As its temperature rises over the constant and uniform room temperature, the body loses energy via conduction (w_{cond}), convection (w_{conv}), and radiation, as a grey body emitter (w_{irr}).

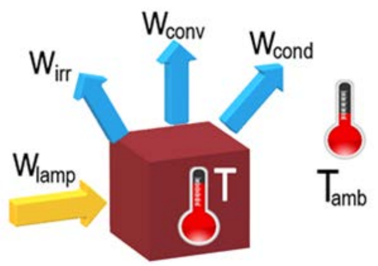

Figure S7. Schematization of the irradiated body. Power flowing in is represented by the yellow arrow, power flowing out by blue arrows.

The net power the body receives is thus:

$$w_{tot} = w_{lamp} - w_{cond} - w_{conv} - w_{irr} \qquad (S1)$$

The conduction and convection terms, w_{cond} and w_{conv}, can in these hypotheses be expressed as directly proportional to the temperature difference between the body and the environment ($\Delta T = T - T_{env}$):

$$w_{conv} + w_{cond} = h_c \Delta T \qquad (S2)$$

Note that in this case, we include in the term h_c also the thermal exchange surface, which is constant for a given setup. The same form can be used for w_{irr}:

$$w_{irr} = A\varepsilon\sigma(T_{env}^2 + T^2)(T_{env} + T)(T - T_{env}) = h_{ir}\Delta T \qquad (S3)$$

Equation S3 establishes $w_{irr} \propto \Delta T = T - T_{env}$, as in the convection/conduction case. This form is not used often, since as it can be clearly seen by its derivation, the term h_{ir} is strongly dependent on temperature and assuming it is a constant leads to greater error than the convection/conduction case. Replacing S3 and S2 in S1 yields

$$w_{tot} = w_{lamp} - h_{con}\Delta T - h_{ir}\Delta T = w_{lamp} - h\Delta T \qquad (S4)$$

To keep the model as simple as possible, we assume the heat exchange coefficient $h(\Delta T) = h_0$. It has been observed that this assumption retains good agreement with experimental data.

The goal is obtaining an equation describing $\Delta T(t)$. Let us consider the first principle of thermodynamics, where m is the mass, c the specific heat and q_{tot} the absorbed heat:

$$\Delta T = \frac{q_{tot}}{mc} \qquad (S5)$$

Since $\frac{dq_{tot}}{dt} = w_{tot}$, deriving both constituents of Equation S5 and then substituting the result into Equation S4 yields a differential equation for $\Delta T(t)$:

$$\frac{d\Delta T}{dt} = \frac{w_{lamp}}{mc} - \frac{h}{mc}\Delta T \qquad (S6)$$

It can be easily verified that the solution of Equation S6, given the boundary condition $T(0) = T_{env}$, is an asymptotic, exponential-regulated growth:

$$\Delta T(t) = \Delta T_{\infty}\left(1 - e^{-\frac{t}{\tau}}\right) \qquad (S7)$$

Where

$$\tau = \frac{mc}{h} \quad e \quad \Delta T_{\infty} = \frac{w_{lamp}}{h} \qquad (S8)$$

S8. TRANSIENT BEHAVIOR IN THERMAL EXPERIMENTS - WATER SAMPLES

As stated in the article, the heating curves for water samples illuminated by the incandescent lamp did not fit well with Equation S7. The first 200-300 seconds of the curves in Figure 5c showed an unusual transient behavior, as reported in the magnification in Figure S8. A starting positive concavity gradually transitioning to the negative concavity characteristics of Equation S7 is observed.

A possible explanation for this effect is that the incandescent lamp is highly emissive in a region where water is highly absorbing, i.e., wavelengths longer than 1200 nm. This means that lot of light is absorbed by water on the front side of the container, which theoretically heats up first. Then, heat needs time to travel towards the thermocouple. This should justify the efficacy of the addition of a delay term to Equation S7 in predicting the behavior after the first 300 s.

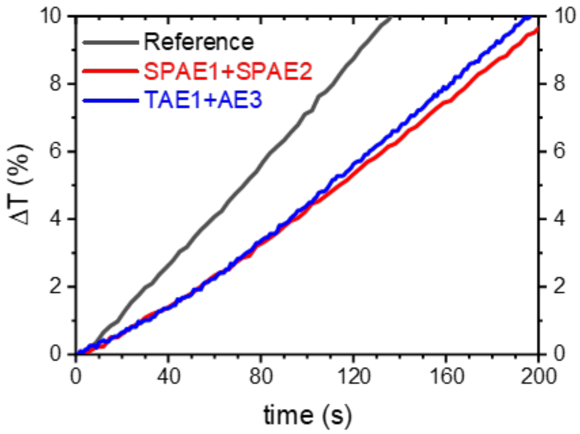

Figure S8. First 200 s of water heating curves from Figure 3b.

References

1. Reference Air Mass 1.5 Spectra. https://www.nrel.gov/grid/solar-resource/spectra-am1.5.html (accessed 2021-10-14).

2. Lova, P.; Manfredi, G.; Comoretto, D., Advances in Functional Solution Processed Planar 1d Photonic Crystals. *Adv. Opt. Mater.* **2018,** *6* (24), 1800730. DOI: 10.1002/adom.201800730

3. Frezza, L.; Patrini, M.; Liscidini, M.; Comoretto, D., Directional Enhancement of Spontaneous Emission in Polymer Flexible Microcavities. *J. Phys. Chem. C* **2011,** *115* (40), 19939-19946. DOI: 10.1021/jp206105r
